# Supplementary material for: SET-9 and SET-26 are H3K4me3 readers and play critical roles in germline development and longevity
Source: eLife. 2018 May 1;7:e34970. doi: 10.7554/eLife.34970 (PMC6010342; doi:10.7554/eLife.34970)
Supplement: Supplementary file 1. — Table S1 (A) Lifespan data for Figure 1B were shown. (B) Heat stress resistance data for Figure 1C were shown. Table S2 (A) Brood size data for Figure 2A were shown. (B) Mortal germline assay data for Figure 2B were show. (C) Maternal and paternal effect on brood size for Figure 2C were shown. (D) Mitotic germ cell number for Figure 2D were shown. Table S3 (A) Wild type lifespan data with RNAi treatment for Figure 3B were shown. (B) rrf-1(pk1417) mutant lifespan data with RNAi treatment for Figure 3C were shown. Table S4 (A) Lifespan data for Figure 3—figure supplement 1A were shown. (B) Heat stress resistance data for Figure 3—figure supplement 1B were shown. (C) Brood size data for Figure 3—figure supplement 1C were shown. Table S5 (A) Histone peptide array binding assay for Figure 6B were shown. Table S6 (A) qPCR RNA-seq validation for set-26(tm2467) compare with wild type data were shown. (B) qPCR RNA-seq validation for F3 set-9(rw5) set-26(tm2467) compare with wild type data were shown. [file elife-34970-supp1.docx]

Table S1

A

Replicate 1

| Strains | # of worms | Mean +/- SD | p value vs N2 | p value vs set-26 |
| --- | --- | --- | --- | --- |
| N2 | 46/57 | 16.96 +/- 0.35 |  |  |
| *set-9* | 48/60 | 17.27 +/- 0.37 | 1 |  |
| *set-26* | 57/66 | 20.60 +/- 0.48 | 3.80E-08 |  |
| *set-9 set-26* | 59/66 | 21.25 +/- 0.46 | 0.00E+00 | 1 |

Replicate 2

| Strains | # of worms | Mean +/- SD | p value vs N2 | p value vs *set-26* |
| --- | --- | --- | --- | --- |
| N2 | 52/65 | 18.62 +/- 0.25 |  |  |
| *set-9* | 44/67 | 18.29 +/- 0.43 | 1 |  |
| *set-26* | 43/72 | 21.35 +/- 0.53 | 5.00E-06 |  |
| *set-9 set-26* | 42/67 | 23.14 +/- 0.42 | 0.00E+00 | 0.1797 |

Replicate 3

| Strains | # of worms | Mean +/- SD | p value vs N2 | p value vs *set-26* |
| --- | --- | --- | --- | --- |
| N2 | 69/73 | 17.77 +/- 0.29 |  |  |
| *set-9* | 61/66 | 16.39 +/- 0.27 | 0.0016 |  |
| *set-26* | 38/41 | 20.74 +/- 0.59 | 0.0000024 |  |
| *set-9 set-26* | 71/74 | 22.64 +/- 0.38 | 0 | 0.0554 |

B

Replicate 1

| Strains | # of worms | Mean +/- SD | p value vs N2 | p value vs *set-26* |
| --- | --- | --- | --- | --- |
| N2 | 64 | 11.02 +/- 0.32 |  |  |
| *set-9* | 57 | 11.47 +/- 0.33 | 0.9591 |  |
| *set-26* | 53 | 14.72 +/- 0.43 | 0 |  |
| *set-9 set-26* | 27 | 15.22 +/- 0.59 | 1.2E-08 | 1 |

Replicate 2

| Strains | # of worms | Mean +/- SD | p value vs N2 | p value vs *set-26* |
| --- | --- | --- | --- | --- |
| N2 | 43 | 8.72 +/- 0.19 |  |  |
| *set-9* | 58 | 8.38 +/- 0.20 | 0.7592 |  |
| *set-26* | 63 | 11.29 +/- 0.24 | 0 |  |
| *set-9 set-26* | 57 | 10.95 +/- 0.23 | 6E-09 | 0.785 |

Table S2

A

|  | F1 | F2 | F3 | F4 | F5 | F6 |
| --- | --- | --- | --- | --- | --- | --- |
| N2 | 334 +/- 33 | 340 +/- 32 | 332 +/- 37 | 337 +/- 19 | 346 +/- 26 | 339 +/- 21 |
| *set-9* | 292 +/- 32 | 302 +/- 16 | 293 +/- 31 | 292 +/- 16 | 303 +/- 23 | 299 +/- 24 |
| *set-26* | 283 +/- 29 | 303 +/- 16 | 302 +/- 37 | 291 +/- 18 | 298 +/- 10 | 289 +/- 25 |
| *set-9 set-26* | 213 +/- 36 | 50 +/- 77 | 56 +/- 85 | 45 +/- 68 | 37 +/- 70 | 26 +/- 48 |

B

|  | F1 | F2 | F3 | F4 | F5 | F6 |
| --- | --- | --- | --- | --- | --- | --- |
| N2 | 100% | 100% | 100% | 100% | 100% | 100% |
| *set-9* | 100% | 100% | 100% | 100% | 100% | 100% |
| *set-26* | 100% | 100% | 100% | 100% | 100% | 100% |
| *set-9 set-26* | 100% | 76% | 52% | 28% | 12% | 0% |

C

| Progeny | Mean +/- SD |
| --- | --- |
| Progeny from P0 male X P0 herm | 165 +/- 38 |
| Progeny from F1 male X P0 herm | 163 +/- 39 |
| Progeny from F1 male X F1 herm | 52 +/- 68 |
| Progeny from P0 male X F1 herm | 51 +/- 63 |

D

| Strain | Mean +/- SD |
| --- | --- |
| N2 | 157 +/- 2.51 |
| F1 *set-9 set-26* | 65 +/- 7.4 |
| F2 *set-9 set-26* | 52 +/- 3.6 |
| F3 *set-9 set-26* | 49 +/- 3.7 |
| F4 *set-9 set-26* | 53 +/ -6.2 |
| F5 *set-9 set-26* | 55 +/- 4.6 |
| F6 *set-9 set-26* | 52 +/- 4.2 |

Table S3

A

| Strains | # of worms | Mean +/- SD | p value vs N2+L4440 |
| --- | --- | --- | --- |
| N2+L4440 | 65/70 | 18.18 +/- 0.41 |  |
| N2*+set-9/26* RNAi | 66/70 | 21.49 +/- 0.50 | 0.0000012 |
| N2*+wdr-5.1* RNAi | 64/67 | 21.63 +/- 0.61 | 0.00000066 |

| Strains | # of worms | Mean +/- SD | p value vs N2+L4440 |
| --- | --- | --- | --- |
| N2+L4440 | 62/67 | 16.61 +/- 0.52 |  |
| N2*+set-9/26* RNAi | 64/67 | 20.00 +/- 0.55 | 0.0001 |
| N2*+wdr-5.1* RNAi | 68/72 | 20.76 +/- 0.56 | 0.00000099 |

B

| Strains | # of worms | Mean +/- SD | p value vs rrf-1+L4440 |
| --- | --- | --- | --- |
| *rrf-1*+L4440 | 56/70 | 16.84 +/- 0.47 | n.a |
| *rrf-1+set-9/26* RNAi | 59/70 | 16.50 +/- 0.46 | 1 |
| *rrf-1+wdr-5.1* RNAi | 60/69 | 20.32 +/- 0.68 | 0.0000085 |

| Strains | # of worms | Mean +/- SD | p value vs rrf-1+L4440 |
| --- | --- | --- | --- |
| *rrf-1*+L4440 | 59/67 | 14.79 +/- 0.44 | n.a |
| *rrf-1+set-9/26* RNAi | 59/66 | 15.43 +/- 0.46 | 0.6413 |
| *rrf-1+wdr-5.1* RNAi | 61/67 | 17.58 +/- 0.56 | 0.0004 |

Table S4

A

| Strains | # of worms | Mean +/- SD | p value vs N2 | p value vs *set-26* |
| --- | --- | --- | --- | --- |
| N2 | 45/58 | 17.51 +/- 0.33 |  |  |
| *set-26* | 55/61 | 20.62 +/- 0.46 | 0.00000075 |  |
| *set-26::gfp* | 52/61 | 18.77 +/- 0.37 | 0.0195 | 0.0024 |

| Strains | # of worms | Mean +/- SD | p value vs N2 | p value vs *set-26* |
| --- | --- | --- | --- | --- |
| N2 | 35/49 | 17.71 +/- 0.35 |  |  |
| *set-26* | 70/86 | 20.88 +/- 0.42 | 0.000000054 |  |
| *set-26::gfp* | 53/80 | 19.25 +/- 0.34 | 0.0042 | 0.0006 |

B

| Strains | # of worms | Mean +/- SD | p value vs N2 | p value vs *set-26* |
| --- | --- | --- | --- | --- |
| N2 | 61 | 7.02 +/- 0.15 |  |  |
| *set-26* | 60 | 7.97 +/- 0.19 | 0.0002 |  |
| *set-26::gfp* | 52 | 7.15 +/- 0.19 | 1 | 0.0065 |

| Strains | # of worms | Mean +/- SD | p value vs N2 | p value vs *set-26* |
| --- | --- | --- | --- | --- |
| N2 | 67 | 7.01 +/- 0.14 |  |  |
| *set-26* | 56 | 7.86 +/- 0.18 | 0.0008 |  |
| *set-26::gfp* | 55 | 7.24 +/- 0.20 | 0.5832 | 0.0691 |

C

| Strain | Mean +/- SD |
| --- | --- |
| N2 | 294 +/- 24 |
| F3 *set-9 set-26* | 39 +/- 60 |
| *set-9::gfp set-26::gfp* | 242 +/- 20 |

Table S5

A

| Peptide# | Mean +/- SD |
| --- | --- |
| P22 | 53716 +/- 14910 |
| P24 | 43989 +/- 18399 |
| P19 | 41971 +/- 35922 |
| P25 | 33272 +/- 13841 |
| P23 | 32016 +/- 16214 |
| P21 | 21673 +/- 20341 |
| P20 | 18864 +/- 24133 |
| P148 | 11574 +/- 14806 |
| P38 | 9542 +/- 10432 |
| H3K4me3 | 0 +/- 0 |
| H3K9me3 | 0 +/- 0 |

Table S6

A

|  | *tiar-2* | *vit-1* | *Y43D4A.5* | *bli-2* | *wrt-2* | *cah-1* | *grd-11* | *nhr-172* | *bath-3* | *F14D2.11* |
| --- | --- | --- | --- | --- | --- | --- | --- | --- | --- | --- |
| qPCR | -2.1 | -2.6 | -1.5 | -2.2 | -1.6 | 2.7 | 3.1 | 2.0 | 1.6 | 1.8 |
| RNA-seq | -2.9 | -3.1 | -2.0 | -2.2 | -1.6 | 2.9 | 3.1 | 3.0 | 2.8 | 1.7 |

B

|  | *asp-12* | *M04C3.1* | *grd-13* | *fipr-2* | *fbn-1* | *his-1* | *catp-3* | *inx-2* | *che-3* | *gst-24* |
| --- | --- | --- | --- | --- | --- | --- | --- | --- | --- | --- |
| qPCR | -1.8 | -1.6 | -2.3 | -2.0 | -1.9 | 2.0 | 2.9 | 2.3 | 2.6 | 2.3 |
| RNA-seq | -2.2 | -2.0 | -2.0 | -2.0 | -1.9 | 2.4 | 2.0 | 2.9 | 1.9 | 1.9 |
